# Structural insights into HIV-1 polyanion-dependent capsid lattice formation revealed by single particle cryo-EM

**DOI:** 10.1073/pnas.2220545120

**Published:** 2023-04-24

**Authors:** Carolyn M. Highland, Aaron Tan, Clifton L. Ricaña, John A. G. Briggs, Robert A. Dick

**Affiliations:** ^a^Department of Molecular Biology and Genetics, Cornell University, Ithaca, NY 14853; ^b^Weill Institute for Cell and Molecular Biology, Cornell University, Ithaca, NY 14853; ^c^Structural Studies Division, Medical Research Council Laboratory of Molecular Biology, Cambridge CB2 0QH, UK; ^d^Department of Cell and Virus Structure, Max Planck Institute of Biochemistry, Munich 82512, Germany

**Keywords:** retrovirus structure, HIV-1, capsid, cryoelectron microscopy, cryoelectron tomography

## Abstract

The HIV-1 capsid houses the viral genome and interacts extensively with host cell proteins throughout the viral life cycle. It is composed of capsid protein (CA), which assembles into a conical fullerene lattice composed of roughly 200 CA hexamers and 12 CA pentamers. Previous structural analyses of individual CA hexamers and pentamers have provided valuable insight into capsid structure and function, but detailed structural information about these assemblies in the broader context of the capsid lattice is lacking. In this study, we combined cryoelectron tomography and single particle analysis (SPA) cryoelectron microscopy to determine structures of continuous regions of the capsid lattice containing both hexamers and pentamers. We also developed a method of liposome scaffold-based in vitro lattice assembly (“lattice templating”) that enabled us to directly study the lattice under a wider range of conditions than has previously been possible. Using this approach, we identified a critical role for inositol hexakisphosphate in pentamer formation and determined the structure of the CA lattice bound to the capsid-targeting antiretroviral drug GS-6207 (lenacapavir). Our work reveals key structural details of the mature HIV-1 CA lattice and establishes the combination of lattice templating and SPA as a robust strategy for studying retroviral capsid structure and capsid interactions with host proteins and antiviral compounds.

## Significance

The mature HIV-1 capsid is composed of the capsid (CA) protein arranged in a conical lattice of hexamers and pentamers. Numerous structures of individual CA hexamers and pentamers alone have been published, but the molecular details of these assemblies in a more global, lattice-wide context are lacking. Here, we present cryoelectron microscopy structures of continuous regions of the capsid lattice containing both hexamers and pentamers. We also describe key differences in the assembly and structures of these oligomers that have important implications for understanding retroviral maturation and for ongoing efforts to pharmacologically target the HIV-1 capsid.

After budding from a cell, immature HIV-1 undergoes maturation to become infectious. In this viral protease-mediated process, the incomplete spherical immature protein lattice of ~400 Gag hexamers is dismantled and replaced by a new, morphologically distinct mature lattice of capsid (CA) molecules proteolytically released from Gag (*SI Appendix*, Fig. S1*A*) (reviewed in refs. 1 and 2). CA assembles into a conical lattice formed by roughly 200 CA hexamers and exactly twelve CA pentamers, the latter of which facilitate lattice curvature and closure around the viral genome (3–5). Monomeric CA consists of two folded domains connected by a flexible linker (6–8): The N-terminal domain (NTD) faces the outer surface of the capsid, where it stabilizes the hexamer, while the C-terminal domain (CTD) forms dimeric interactions that link hexamers together (4, 9, 10). Beyond its structural and genome-protective roles, the capsid also interfaces extensively with host cell proteins, many of which have been co-opted by the virus to ensure its proper trafficking and uncoating within the cell. As such, CA has become the focus of an emerging class of capsid-targeting antiretroviral compounds that interfere with capsid–host factor interactions and compromise capsid structural integrity (reviewed in refs. 11–13).

Most published high-resolution structural information about the mature capsid has come from X-ray crystallography and NMR analyses of individual CA hexamers, pentamers, and monomers. Although these studies have certainly provided invaluable insights into capsid structure (8, 14–18) and its interactions with small molecules (reviewed in ref. 12) and host proteins (6, 19–21), we currently lack detailed structural information about hexamers and pentamers in a global, lattice-wide context. Recent advances in cryoelectron microscopy (cryo-EM) and cryoelectron tomography (cryo-ET) have yielded additional important insights into capsid structure (4, 5, 9, 22, 23), but resolution has generally remained limited, particularly for the pentamer.

Consequently, important questions about capsid structure and assembly remain unanswered. For instance, the polyanionic HIV-1 assembly cofactor inositol hexakisphosphate (IP_6_) is known to coordinate rings of electropositive charge in the central pores of Gag and CA hexamers (24–29), but the role it plays in CA pentamer structure, if any, remains uncertain. Molecular dynamics simulations suggest that IP_6_ coordinates pentamers in a manner similar to its coordination of hexamers (23, 29, 30), but there are no published experimental structural data clearly demonstrating such an interaction. Polyanionic deoxyribonucleotide triphosphates (dNTPs) also interact with and translocate through hexamer central pores (30–32)—an observation with important implications for capsid nucleotide import and reverse transcription—but whether dNTPs, like IP_6_, also have a role in stabilizing capsid structure is unknown.

In this study, we developed an in vitro CA lattice “templating” assembly technique that enabled us to enrich for CA pentamer formation and to use SPA to examine the lattice under a range of assembly conditions. Using this approach, we demonstrated that pentamer formation is strictly dependent on polyanion coordination, providing a key advance in our understanding of capsid assembly and, more broadly, HIV-1 maturation. We also used lattice templating to determine structures of the lattice bound to the first-in-class capsid-targeting antiretroviral compound GS-6207 (lenacapavir). We found that GS-6207 binds exclusively to hexamers, a behavior that arises from key structural differences between pentamers and hexamers. Altogether, this work provides important insights into the molecular mechanisms of HIV-1 capsid assembly, structure, and function.

## Results

### Structure of HIV-1 Capsid-Like Particles (CLPs) Determined via Cryo-ET.

**Fig. 1.**
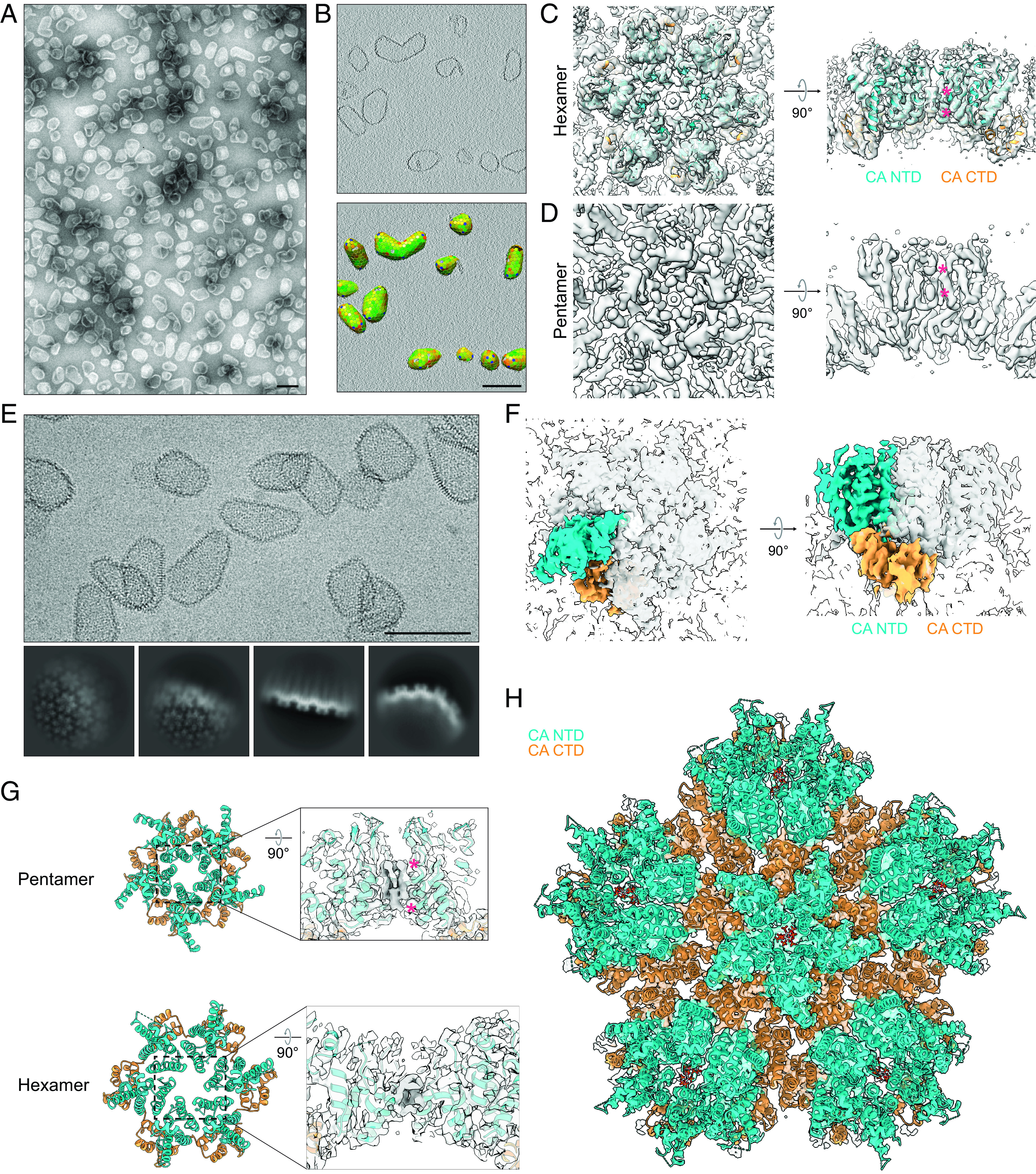
Cryoelectron tomography and single particle cryoelectron microscopy of HIV-1 capsid-like particles. (*A*) Negative stain TEM micrograph of capsid-like particles (CLPs) prepared in vitro from purified CA and IP_6_. (*B*) Central “slice” through CLPs in a tomogram (*Top*) with corresponding lattice maps determined by subtomogram averaging (*Bottom*). Hexamers in the lattice map are colored from green to red according to cross-correlation (CC) with the average hexamer structure, with red indicating lower CC and green indicating higher CC. Pentamers are colored blue. (*C*) Cryo-ET reconstruction of the CLP hexamer (EMD-16699) with crystal structure 4XFX (Gres et al., 15) docked according to best cross-correlation with the cryo-EM density. Densities corresponding to IP_6_ are marked with magenta asterisks. (*D*) Cryo-ET reconstruction of the CLP pentamer (EMD-16698). Densities corresponding to IP_6_ are marked with asterisks. (*E*) Cryo-EM micrograph of CLPs (*Left*) with select two-dimensional (2D) class averages (*Right*). (*F*) Cryo-EM (SPA) map of CLP central pentamer (EMD-29772). Density corresponding to a single CA monomer is highlighted with color. (*G*, *Right*) densities corresponding to IP_6_ (marked with magenta asterisks) in pentamer (*Top*) and hexamer (*Bottom*) cryo-EM maps (EMD-29772). (*Left*) atomic model for CLP pentamer (*Top*) and hexamer (*Bottom*) (PDB 8G6K). (*H*) Atomic model for “global” pentamer structure (pentamer surrounded by its five nearest hexamer neighbors) (PDB 8G6K) with corresponding cryo-EM map (EMD-29772). *myo*-IP_6_ molecules are docked according to best cross-correlation with the cryo-EM density. In all maps and models shown, the CA NTD is shown in cyan, and the CA CTD is shown in orange. Side views in *C*, *D*, and *G* show central pore cross-sections. (Scale bars, 100 nm.)

Although a good general understanding of HIV-1 capsid structure has been available for some years, high-resolution structures of HIV-1 hexamers and pentamers in the context of the mature lattice have not been determined, which limits the understanding of the principles of capsid structure and assembly. We therefore sought to determine structures of these complexes using cryo-ET with subtomogram averaging and single particle analysis (SPA) cryo-EM. As reported previously (25), purified wild-type HIV-1 CA assembles efficiently into CLPs at pH 6.2 in the presence of the essential assembly cofactor IP_6_ (Fig. 1*A* and *SI Appendix*, Fig. S1*B*), which is coordinated within the CA hexamer pore by residues R18 and K25 in α-helix 1 (*SI Appendix*, Fig. S1*A*). These CLPs have a predominantly conical morphology resembling that of authentic HIV-1 capsids (3, 5, 33, 34). The hexamer pore has also been proposed to function as a dNTP import channel (30, 31). We therefore also assessed whether dNTPs stimulate CLP assembly but found that they do not (*SI Appendix*, Fig. S1*B*).

We imaged IP_6_-assembled CLPs by cryo-ET (*SI Appendix*, Table S1 and Fig. S1 *B*–*D*) and determined the structures of their constituent hexamers by subtomogram averaging to an estimated resolution of 3.9 Å. Using the aligned hexamer subtomogram positions, we constructed lattice maps that mark the positions of hexamers and pentamers within the CLPs (Fig. 1*B*). As in authentic HIV-1 capsids (3–5), the conical CLPs consist of a lattice of CA hexamers closed by 12 pentamers. Finally, we applied subtomogram averaging to pentamer positions identified in the lattice maps, resulting in a pentamer structure with an estimated resolution of 6.2 Å (Fig. 1*D*).

The structures of both hexamers and pentamers in the CLPs are virtually indistinguishable from those previously determined at lower resolution from capsids in authentic HIV-1 viral particles (5). We observed strong densities corresponding to established and hypothesized IP_6_ binding sites in the central pores of both hexamers and pentamers (Fig. 1 *C* and *D*) (25, 26, 29), in agreement with a recent low-resolution cryo-ET study of authentic virions (23). While the hexamer structure in published crystal structures is equivalent to that of hexamers within authentic capsids (Fig. 1*C* and refs. 5 and 18), the structure of the pentamer within authentic capsids differs from crystal structures of cross-linked pentamers (*SI Appendix*, Fig. S1*C* and ref. 17), raising the question of whether the internal environment of the virion or other viral components are required to stabilize the “viral” pentamer. Our data demonstrate that a minimal system consisting of only CA and IP_6_ is sufficient to reconstitute the authentic in-virus structures of both the hexamer and the pentamer, as well as the conical fullerene architecture of the capsid.

### Structure of the HIV-1 CLP Lattice Determined via SPA Cryo-EM.

We next sought to generate higher-resolution structures of the CLP pentamer using SPA cryo-EM (*SI Appendix*, Table S1). Lattice details in flat (hexamer rich), curved (pentamer containing), and edge regions were clearly observable in micrographs and in 2D class averages (Fig. 1*E*). We focused our analysis on regions of the lattice containing pentamers and determined the structure of the pentamer surrounded by five hexamers (the “global” pentamer structure) to an estimated resolution of 3.6 Å (*SI Appendix*, Fig. S2 and Fig. 1 *F*–*H*). In accordance with our cryo-ET data (Fig. 1 *C* and *D*), strong densities were present at known R18 and K25 IP_6_ binding sites in the hexamer (25, 26, 29) and at the same sites in the pentamer, confirming molecular dynamics predictions of pentamer IP_6_ binding (23, 29, 30). Importantly, these SPA hexamer and pentamer structures are indistinguishable from those determined via cryo-ET (*SI Appendix*, Fig. S1 *D* and *E*) and via an independent SPA study described in the accompanying manuscript (35), demonstrating that SPA is suitable for structural studies of complex CA lattices containing both hexamers and pentamers.

### HIV-1 CA Lattice Formation via Protein Templating on Lipid Vesicles.

As in isolated authentic capsids (3–5), pentamers in our CLPs were quite scarce, present at a pentamer to hexamer ratio of ~1:20. We therefore wondered whether the resolution of the pentamer map could be further improved by increasing this ratio. Given the relationship between pentamers and lattice curvature, we speculated that pentamers could be enriched by forcing CA to form a high-curvature lattice similar to that permitted by pentamers at the ends of authentic capsid cores (3–5). Indeed, we and others have shown that CA from other retroviruses can be induced to assemble into highly curved icosahedral particles enriched in pentamers under specific buffer and assembly conditions (36–38). For HIV-1, however, this is possible only with the introduction of mutations (39, 40).

**Fig. 2.**
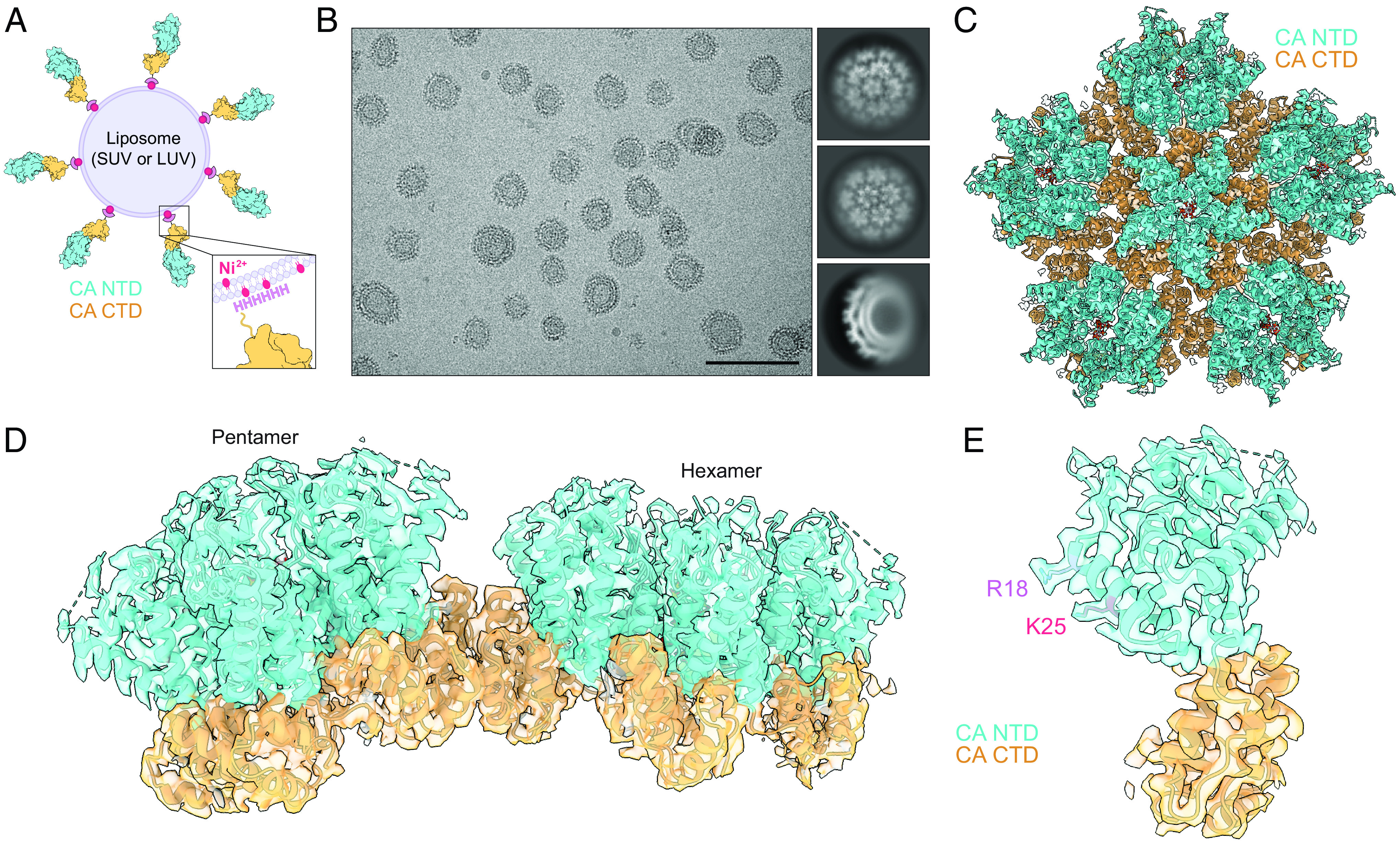
Single particle analysis of liposome-templated HIV-1 CA lattice. (*A*) Diagram of CA lattice templating scheme. Purified CA-6xHis is anchored to liposomes containing nickel-chelated lipids. When applicable, polyanions are then added. SUV, small unilamellar vesicle; LUV, large unilamellar vesicle. (*B*) Cryo-EM micrograph of SUV-templated CA with IP_6_ (pH 7.4) (*Left*) with select 2D class averages (*Right*). (Scale bar, 100 nm.) (*C*–*E*) Atomic model for liposome-templated CA lattice (prepared at pH 7.4 in the presence of IP_6_) (PDB 8G6M) with corresponding cryo-EM map (EMD-29774). *myo*-IP_6_ molecules are docked according to best cross-correlation with the cryo-EM density. The CA NTD is shown in cyan, and the CA CTD is shown in orange. (*C*) Global pentamer structure (pentamer surrounded by its five nearest hexamer neighbors). (*D*) Focused view of adjacent pentamer and hexamer. (*E*) Focused view of a single CA pentamer monomer with IP_6_-coordinating R18 and K25 side chains highlighted in lavender and magenta, respectively.

We therefore devised a lattice assembly scheme in which purified wild-type HIV-1 CA-6xHis is anchored to highly curved ~30-nm small unilamellar vesicle (SUV) scaffolds doped with nickel-chelated lipids (Fig. 2*A*). Importantly, the extreme C terminus of the CA CTD (G222-L231) is not known to make critical contacts for mature lattice formation as this region is disordered past V221 in cryo-EM and crystal structures (5, 18). Curiously, we found that this “SUV-templated” CA forms a lattice that partially covers the SUVs independently of IP_6_ at both pH 6.2 and pH 7.4, while addition of either IP_6_ or dNTPs drives full assembly of the lattice around the SUVs (*SI Appendix*, Fig. S3*A*). By visual inspection, the assembled lattice does not differ between the two pH conditions or in the presence or absence of either IP_6_ or dNTPs, and the lattice generally follows the curvature of the SUVs. We also tested whether a lattice could form on larger ~100-nm large unilamellar vesicles (LUVs) in the presence of IP_6_ and found that it indeed does, but it also appears to deform the liposome membrane at sites of pentamer incorporation (*SI Appendix*, Fig. S3 *B* and *C*). These results indicate that CA can be induced to form a highly curved lattice and that unlike CLP assembly, formation of the SUV-templated CA lattice is much less dependent on pH or the presence of IP_6_.

### Structures of SUV-Templated CA Lattice Determined via SPA Cryo-EM.

To determine whether SUV-templated CA lattice is comparable to that of CLPs and authentic capsids, we determined the structures of SUV-templated CA lattice in the presence of IP_6_ at pH 6.2 (*SI Appendix*, Fig. S4) and at pH 7.4 (Fig. 2 *B*–*E* and *SI Appendix*, Fig. S4) by SPA (*SI Appendix*, Fig. S2). The estimated resolutions for the global pentamer structures were 3.3 Å and 3.1 Å, respectively. As predicted, the increased lattice curvature results in a significant increase in the pentamer to hexamer ratio, from ~1:20 to ~1:5 (*SI Appendix*, Fig. S3*D*), suggesting that fewer hexamers are needed to form a complete lattice around the highly curved SUVs. This is similar to highly curved regions of authentic capsids, which contain fewer hexamers per pentamer than do flatter regions of the lattice (5). By comparison, the pentamer to hexamer ratio in LUV-templated lattice is ~1:20, reminiscent of the ratio found in our CLPs and in authentic virions (3–5).

The global pentamer structures are virtually indistinguishable between CLP and SUV-templated lattice preparations (*SI Appendix*, Fig. S4). This observation, along with the ability of templated CA to accommodate a broader range of assembly conditions, indicates that liposome templating is a useful tool for structural studies of the lattice under more physiological buffer conditions than have previously been feasible for HIV-1 CA. In addition, the curvature imposed on the CA lattice by templating on SUVs or LUVs does not alter the native geometry of the CA pentamer or its nearest hexamer neighbors, highlighting the strict local lattice curvature imposed by the pentamer [*SI Appendix*, Figs. S4*A* and S3*C*; see also accompanying manuscript (35)].

### Pentamer Formation Is Dependent on Polyanion Coordination.

**Fig. 3.**
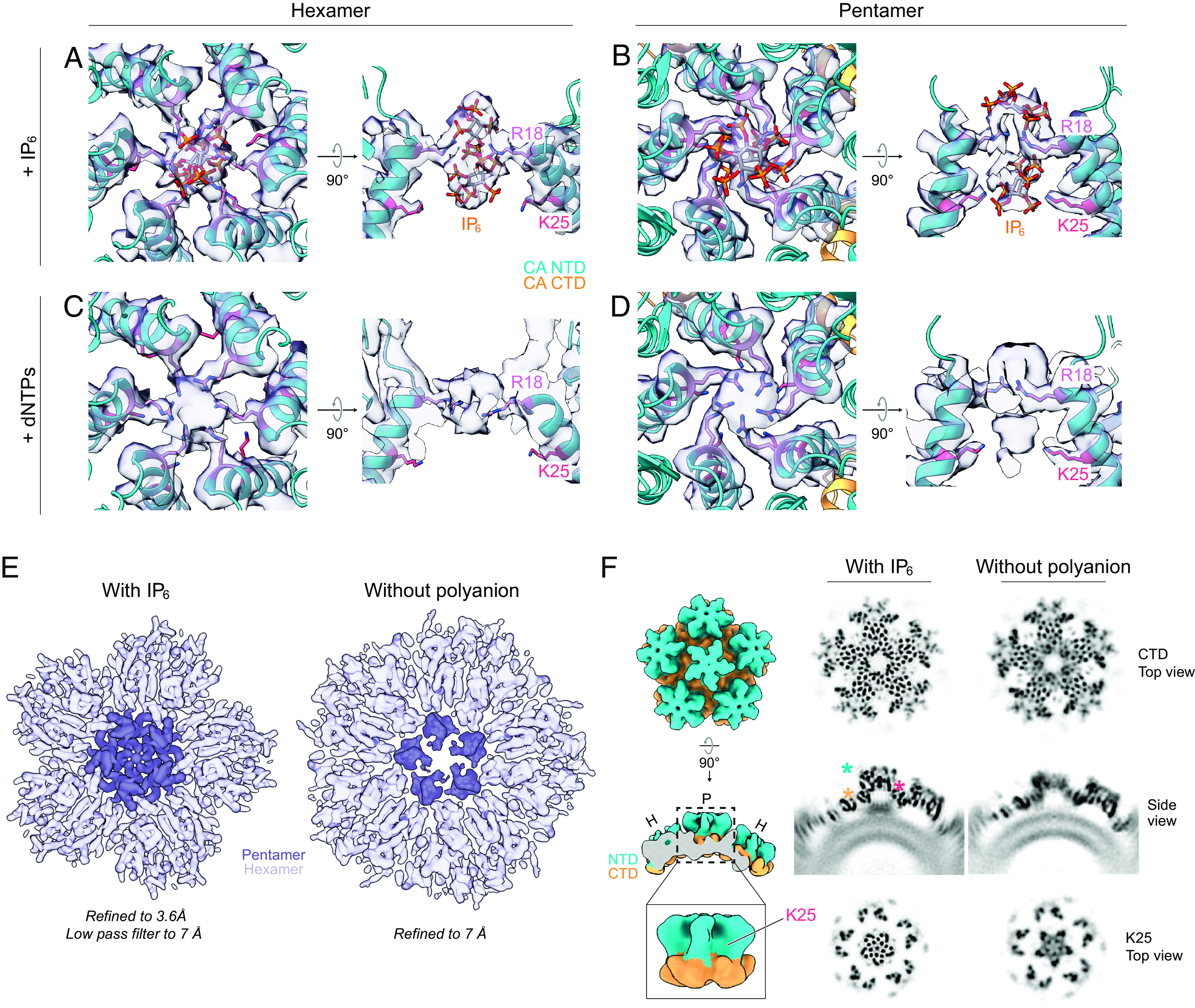
Polyanions are required for HIV-1 CA pentamer formation. (*A*–*D*) Atomic models and corresponding cryo-EM densities of polyanions coordinated in CA hexamer (*A* and *C*) and pentamer (*B* and *D*) central pores. Lattice was prepared via liposome templating of CA-6xHis at pH 7.4 in the presence of IP_6_ (*A* and *B*) (EMD-29774, PDB 8G6M) or dNTPs (*C* and *D*) (EMD-29775, PDB 8G6N). In *A* and *B*, *myo*-IP_6_ molecules are docked according to best cross-correlation with the cryo-EM density. For *C* and *D*, hypothesized dNTP orientations are shown in *SI Appendix*, Fig. S4*A*. In all *A*–*D* side views, helices from only two CA monomers are shown for clarity. (*E*) Global cryo-EM maps of templated HIV-1 CA-6xHis lattice prepared at pH 7.4 in the presence or absence of IP_6_. Pentamer (*Left*) and aberrant pentamer (*Right*) regions are highlighted with a darker color. To ensure a fair comparison with the “assembled without polyanion” map (*Right*; EMD-29777), the “assembled with IP_6_” map (*Left*) (EMD-29774) was prepared from a subset of ~11,000 particles randomly selected from the dataset used to determine the structure presented in Fig. 2 and was low pass filtered to 7 Å. (*F*, *Left*) diagram of regions of interest shown in orthoslice views at right. P, pentamer; H, hexamer. (*Right*) orthoslice side and top views of the global cryo-EM maps shown in *E*. Top views show the region containing pentamer K25 or pentamer CTDs. Asterisks mark positions of CA residue K25 (magenta), the CA NTD (cyan), and the CA CTD (orange). Density is shown in black to differentiate it from 2D class averages. For the models in *A*–*D* and diagram in *F*, the CA NTD is shown in cyan, and the CA CTD is shown in orange.

We next sought to understand the role of IP_6_ in CA lattice formation. In agreement with the CLP data (Fig. 1 *C*, *D*, and *G*), we noted strong densities corresponding to two established IP_6_ binding sites in hexamer central pores from the SUV-templated CA lattice prepared at pH 7.4 (25, 26, 30): a primary binding site above the R18 ring and a secondary site near K25 below the ring (Fig. 3*A*). We also observed two densities at similar positions within the central pore of the pentamer, although the contribution of K25 to IP_6_ coordination appeared stronger in the pentamer than in the hexamer (Fig. 3 *A* and *B*). We noted the same features in lattice assembled in the presence of dNTPs instead of IP_6_ (Fig. 3 *C* and *D*) (global pentamer map estimated resolution of 3.5 Å; *SI Appendix*, Fig. S2), indicating that both polyanions can drive formation of hexamers and pentamers under these conditions. We were unable to determine dNTP orientation or number within either oligomer, but we suggest some possibilities based on published molecular dynamics simulations (30) in *SI Appendix*, Fig. S5*A*.

Next, we compared the cryo-EM dataset used to generate the IP_6_-containing CA lattice structure presented in Fig. 2 and in Fig. 3 *A* and *B* to a dataset containing SUV-templated CA lattice in the absence of any polyanion (Fig. 3 *E* and *F* and *SI Appendix*, Fig. S5 *B* and *C*). Intriguingly, while hexamers assembled normally in the absence of polyanion, pentamers were severely defective. They were also rarer than in lattice assembled with polyanions: The ratio of these aberrant “pseudopentamers” to hexamers was ~1:10, in contrast to the 1:5 ratio seen in SUV-templated CA lattice assembled with polyanion. We generated a pseudopentamer global structure refined to an estimated resolution of 7.1 Å and found that the CTDs of CA monomers appear fairly normal, while the NTDs are largely unresolved (*SI Appendix*, Fig. S5*D*). These data suggest that pentamer formation is strictly dependent on coordination of polyanions such as IP_6_, while hexamer formation is not under these conditions. Indeed, hexamer pores in our no-polyanion SUV-templated CA lattice dataset contain a density much smaller than that that of IP_6_ (*SI Appendix*, Fig. S5*B*), which we hypothesize corresponds to a small anion that helps neutralize the R18 charge (41), allowing hexamers to form.

To assess the importance of polyanion coordination in vivo, we generated several R18 (Gag R150) and K25 (Gag K157) CA mutants and measured their infectivity and release in tissue culture (*SI Appendix*, Fig. S5 *F*–*H*). In agreement with published studies (25, 29, 31, 42) all mutants had drastically reduced infectivity, likely due to decreased virus-like particle (VLP) release from infected cells. Mutations to K25 have previously been shown to also have deleterious effects on capsid morphology and stability (28, 29), so we also examined the capacity of purified CA^K25N^ to assemble in the presence of IP_6_ (*SI Appendix*, Fig. S5*E*). Intriguingly, we found that it forms mostly tubular assemblies, which are known to contain only hexamers (4, 9). This result is consistent with the tendency of purified HIV-1 CA to form tubes, but not polyhedrons, in the absence of IP_6_ under high-salt (1 M NaCl) conditions (10, 43, 44). We propose that a major role of IP_6_ in HIV-1 virion production is to drive pentamer formation and, thus, complete assembly of the capsid around the viral genome.

### Lenacapavir Selectively Binds to the CA Hexamer.

**Fig. 4.**
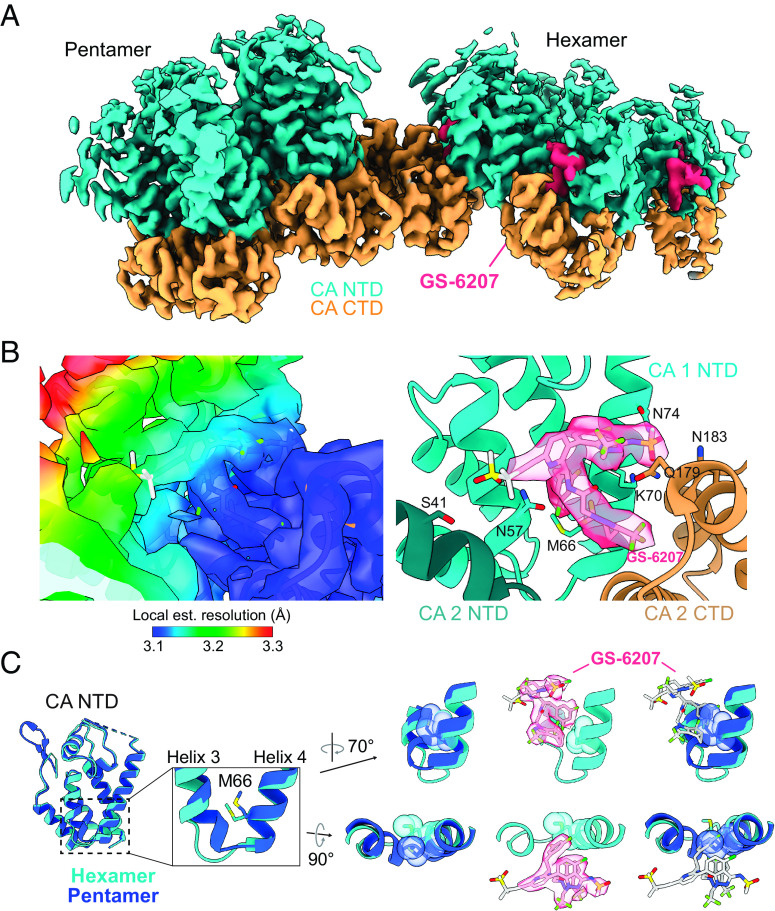
Lattice templating reveals new insights into GS-6207 binding. (*A*) Cryo-EM map of GS-6207 bound to the CA lattice (EMD-29776). Lattice was prepared via SUV templating of CA-6xHis at pH 7.4 in the presence of IP6. GS-6207 density is shown in magenta. (*B*) A GS-6207 binding pocket between two adjacent CA monomers from the structure in *A*. (*Left*) Cryo-EM map colored by local estimated resolution; GS-6207 and atomic model (PDB 8G6O) docked according to best cross-correlation with the cryo-EM density. (*Right*) Atomic model with GS-6207 docked according to best cross-correlation with the cryo-EM density. CA residues that have been reported to interact with GS-6207 are labeled. (*C*) Comparison of hexamer (cyan) and pentamer (blue) CA NTDs from the structure shown in *A*. (*Left*) overlaid hexamer and pentamer NTDs. (*Middle*) M66 “gating” residue in the hexamer–pentamer switch region between ɑ-helices 3 and 4. (*Right*) View of the hexamer CA NTD GS-6207 binding site compared with the same region for the pentamer. GS-6207 is docked according to best cross-correlation with the cryo-EM density. For the map shown in *A* and the model shown in *B*, the CA NTD is shown in cyan, and the CA CTD is shown in orange.

Finally, we extended our lattice templating approach to understanding the interaction of the capsid-targeting antiretroviral compound GS-6207 (lenacapavir; marketed by Gilead Sciences as Sunlenca®) with CA. We determined the structure of this compound bound to SUV-templated CA lattice at an estimated resolution of 3.1 Å. Consistent with crystal structures of cross-linked CA hexamers bound to GS-6207 (45, 46), we found that the compound binds to the ‘FG binding pocket’ between the NTD and CTD of adjacent hexamer monomers (Fig. 4 *A* and *B* and *SI Appendix*, Fig. S2). Importantly, this site is also targeted by FG motif-containing host cell proteins CPSF6, Nup153, and Sec24C (19–21, 35, 45–47). GS-6207 density was completely absent from the pentamer, indicating that the compound binds exclusively to hexamers. This selectivity appears to arise from spatial constraints imposed by subtle conformational differences between pentameric and hexameric CA as the FG binding pocket is altered in the pentamer by a structural “switch” between NTD ɑ-helices 3 and 4. (For more detailed structural comparisons, see refs. 35 and 40) This switch alters the position of M66 in the pentamer such that it would sterically interfere with the difluorobenzyl group of GS-6207 (Fig. 4*C*), providing an explanation for the selective binding of GS-6207 to hexamers.

## Discussion

Application of cryo-ET with subtomogram averaging to our in vitro CLP assembly system confirmed that the distributions and structures of hexamers and pentamers in CLPs match those previously described at lower resolution for authentic HIV-1 capsids (5, 23, 33, 34). SPA cryo-EM of these CLPs [see also accompanying manuscript (35) and recent work from ref. 40] enabled us to collect larger datasets than feasible with cryo-ET, resulting in higher-resolution structures. This was particularly advantageous for the rarer pentamer. The structure of the pentamer within the capsid/CLP lattice differs significantly from published crystal structures of cross-linked pentamers (17) and instead matches the structure of the pentamer within the virion. This indicates that a combination of capsid protein (CA) and IP_6_ is sufficient to reconstitute the viral pentamer structure—no other virus-specific factor is required.

Although our CLP assembly scheme recapitulates the morphology and composition of authentic capsids, it only works at low-pH (pH 6.0 to 6.2) conditions. This is not ideal for studies of capsid interactions with host cell factors and potential capsid-targeting antiretroviral compounds, which, in nature, would interact with the capsid at a physiological pH. The liposome scaffold-based CA lattice templating technique we introduce here enabled us to overcome this limitation and to study lattice assembled under more physiological buffer conditions. A second advantage of the lattice templating system is that it itself promotes lattice formation, likely by simply increasing proximity of CA monomers: We found that SUV-templated CA readily forms some degree of lattice in the absence of typical drivers of in vitro CA lattice assembly such as the essential cofactor IP_6_, which promotes conical CLP and capsid formation (25, 29), or high salt concentration (1 M NaCl), which promotes formation of CA tubes composed exclusively of hexamers (4, 9, 10, 43, 44).

Close inspection of such lattice by cryo-EM revealed that CA hexamers can form normally under these conditions, but pentamers cannot. Our structural data indicate that K25 is particularly important for IP_6_ coordination in the pentamer, and we found that purified mutant CA^K25N^ cannot assemble into conical CLPs, even in the presence of IP_6_, but instead forms hexamer-rich tubes. This suggests that polyanions—specifically IP_6_, given its established importance for virus production and capsid stabilization—are indispensable for pentamer formation. In recent years, IP_6_ has been shown to be important also for lentiviruses equine infectious anemia virus, simian immunodeficiency virus, feline immunodeficiency virus, and bovine immunodeficiency virus (27, 42, 48) and the alpharetrovirus Rous sarcoma virus (38). Given this, and the conservation of regularly spaced Arg or Lys residues in ɑ-helix 1 of CA (49), we hypothesize that the reported polyanion requirement for pentamer formation for HIV-1 is conserved for other retroviruses as well.

A recent molecular dynamics simulation analysis of capsid assembly (50) lends further support to this model, suggesting that IP_6_ is critical for assembly of a high-curvature lattice and that it occupies a greater fraction of pentamers than of hexamers within the lattice. Moreover, a cryo-ET analysis (23) of capsid cores from native virions reported that the central pores of viral pentamers contained two strong densities that were predicted to be IP_6_. Hexamer pores contained much weaker density, although exposure of the capsid cores to excess IP_6_ yielded hexamer structures with clearer densities. The number and mobility of IP_6_ molecules coordinated by capsid CA in nature remains an area of active research (23, 30, 50, 51), but our work suggests that IP_6_ coordination within the pentamer is likely quite stable. We also found that dNTPs can substitute for IP_6_ to drive full assembly of SUV-templated CA lattice. Capsid hexamer pores are thought to facilitate nucleotide import for reverse transcription (30, 31), but whether pentamer pores can also import nucleotides is unknown. Furthermore, whether nucleotides, like IP_6_, have a role in physiological capsid stabilization remains to be determined.

Finally, we determined the structure of SUV-templated CA lattice bound to the potent antiretroviral compound GS-6207 and found that the compound binds exclusively to hexamers at the “FG” binding site between neighboring CA monomers. We attribute this selective binding to a structural “switch” in the CA NTD that promotes a conformational change in CA monomers and drives oligomerization to either the pentameric or the hexameric state (40 and 35). This conformational change reshapes the FG binding pocket in pentamers, resulting in a steric block of GS-6207 access to the site. Work described in the accompanying manuscript demonstrates how this remodeling also prevents binding of FG motif-containing host cell proteins CPSF6, Nup153, and Sec24C to pentamers (35). These findings have important implications for understanding how the HIV-1 capsid interacts with host cell proteins and for ongoing efforts to pharmacologically target the capsid.

Altogether, our work provides key insights into HIV-1 capsid structure and assembly and demonstrates that SPA cryo-EM is useful for structural studies of irregular (i.e., nonicosahedral or tubular) retroviral lattices. In addition, the CA lattice templating method we introduce here has proven to be a valuable platform for interrogating the molecular mechanisms underlying lattice assembly and lattice interactions with small molecules and proteins of interest. We expect that this technique can be extended to studies of other viruses as well.

## Materials and Methods

### Protein Expression and Purification.

Both untagged HIV-1 CA and HIV-1 CA-_6x_His were produced in BL21 *Escherichia coli* cells. Cells were grown to OD_600_ ∼0.8 before induction with 400 uM isopropyl β-d-1-thiogalactopyranoside at 30 °C for 6 h and then collected by centrifugation. Cells were resuspended in lysis buffer (for untagged CA, 25 mM Tris, pH 8.0, 2 mM phenylmethylsulfonyl fluoride (PMSF), 4 mM tris(2-carboxyethyl)phosphine (TCEP); for CA-_6x_His, 50 mM Tris, pH 8.0, 300 mM NaCl, 10 mM imidazole, 2 mM PMSF, and 4 mM TCEP) and lysed by sonication. Insoluble material was collected by ultracentrifugation and discarded. Polyethylenimine was added to the cleared lysate to 0.3% and mixed at 4 °C for 10 min. Bulk protein was then precipitated from the lysate with 20% ammonium sulfate (incubated, stirring, at 4 °C for 40 min), and precipitated protein was collected by centrifugation. The protein pellet was resuspended in CA lysis buffer and then run through a HiPrep desalting column (GE Healthcare Life Sciences) with 50 mM Tris, pH 8.0, and 2 mM TCEP. Protein peak fractions were collected and combined.

For purification of untagged CA and CA^K25N^, CA was isolated from the desalted crude protein via a tandem HiTrap Q HP-HiTrap SP HP anion–cation exchange column setup: Crude protein was applied to the columns (Q first, followed immediately by SP), and flow-through containing CA was collected. Residual protein was collected by washing the column once with 25 mM Tris, pH 8, 2 mM TCEP and then twice with 25 mM Tris, pH 8, 25 mM NaCl, and 2 mM TCEP. Eluate and washes were combined, and buffer exchanged into 25 mM Tris, pH 8 and 2 mM TCEP via gel filtration (Superdex 75 Increase 10/300; GE Healthcare Life Sciences). Protein peak fractions were combined and concentrated to 1 mM, then flash-frozen in liquid nitrogen, and stored at −80 °C.

For purification of CA-_6x_His, NaCl and imidazole were added to the desalted crude protein to final concentrations of 300 mM and 10 mM, respectively. The solution was then incubated with NiNTA agarose resin (Qiagen) for 1 hr at 4°C, rotating. The resin was washed 1x in batch with 20 mL CA-His lysis buffer, then transferred to a gravity column, and washed 10x with 1 mL CA-His wash buffer (50 mM Tris, pH 8.0, 300 mM NaCl, 30 mM imidazole, and 2 mM TCEP). CA-_6x_His was then eluted in 1 mL fractions with CA-His elution buffer (50 mM Tris, pH 8.0, 300 mM NaCl, 300 mM imidazole, and 2 mM TCEP). CA-_6x_His was further purified by gel filtration (Superdex 75 Increase 10/300; GE Healthcare Life Sciences) in CA-His gel filtration buffer (25 mM Tris, pH 8.0, and 2 mM TCEP). Protein peak fractions were combined and concentrated to 500 uM, then flash-frozen in liquid nitrogen, and stored at −80 °C.

### In Vitro CA CLP Assembly.

CLP assembly reactions were buffered with 50 mM MES, pH 6.2, and contained 500 uM purified untagged HIV-1 CA, 2.5 mM IP_6_ (Tokyo Chemical Company), and 1 mM TCEP. CA was first warmed at 37 °C for 5 min, then combined with IP_6_, and incubated at 37 °C for 15 min. Assemblies were stored on ice or at 4 °C until grid preparation. Templated CA lattice assemblies were screened by negative stain transmission electron microscopy (TEM) (FEI Tecnai 12 BioTwin TEM or FEI Morgagni TEM; see Negative Stain EM in the *Materials and Methods* section) before cryogrid preparation.

### Liposome Preparation.

Chloroform stock solutions of 1,2-dioleoyl-sn-glycero-3-phosphocholine (DOPC) and 1,2-dioleoyl-sn-glycero-3-[(N-(5-amino-1-carboxypentyl)iminodiacetic acid)succinyl], nickel salt (DGS-NiNTA) were purchased from Avanti Polar Lipids. Cholesterol was purchased from Nu-Chek Prep (Elysian, MN), and cholesterol stock solutions were prepared by standard gravimetric procedures to within 0.2% error. Stock solutions were mixed at an 85:10:5 DOPC:DGS-NiNTA:cholesterol ratio and then exchanged into 50 mM MES with 2 mM TCEP (pH 6.2 or 7.4) via rapid solvent exchange to a final total lipid concentration of 13 mM. SUVs were prepared by sonication in a room temperature water bath for 30 min (Sonblaster Series 200, Narda Ultrasonics Corporation) and then filtered through a desalting spin column (Thermo Fisher Scientific). LUVs were prepared by extrusion through a 100-nm polycarbonate filter using a miniextruder (Avanti Polar Lipids) 25 times at room temperature.

### CA Lattice Templating on Liposomes.

Purified HIV-1 CA-_6x_His was combined with liposomes and incubated at 37 °C for 5 min. 10 mM IP_6_ (Tokyo Chemical Company) in 50 mM MES (pH 6.2 or 7.4) was added to a final concentration of 1 mM, and the assembly reaction was incubated at 37 °C for 15 min. SUV assembly reactions (pH 6.2 or 7.4) contained 330 µM CA-_6x_His and 5.9 µM SUVs; LUV assembly reactions (pH 7.4) contained 190 mM CA-_6x_His and 3.7 mM LUVs. If applicable, dNTPs (equimolar mixture of dATP, dCTP, dGTP, and dTTP; Cytiva) were added to a final concentration of 2 mM in place of IP_6_. For preparation of GS-6207-bound lattice, GS-6207 (Gilead Sciences) was added to a final concentration of 330 µM 5 min after IP_6_ addition, and the reaction contained 125 mM NaCl and was buffered with 50 mM HEPES (pH 7.4) rather than MES. Templated lattice assemblies were screened by negative stain TEM (FEI Tecnai 12 BioTwin TEM; see Negative Stain EM in the *Materials and Methods* section) before grid preparation.

### Virus Production and Infectivity Assay.

HIV-1^ΔEnv^ consisted of NL4-3-derived proviral vector with a 5′ cytomegalovirus-driven green fluorescent protein and defective for Vif, Vpr, Nef, and Env (kindly provided by Vineet Kewal-Ramani, National Cancer Institute-Frederick). CA mutations were made using the In-Fusion Cloning System (Takara Bio) using either custom Gene Blocks (Integrated DNA Technologies) (R18A, R18L, R18K, K25A, K25E, K25R, K25N, and K25N-N21K) or PCR site-directed mutagenesis (PR-D25A). All plasmids were verified by sequencing. The plasmid for vesicular stomatitis virus glycoprotein (VSV-g, NIH AIDS Reagent Program) has been previously described (52).

HIV-1 VLPs were produced by Lipofectamine 3000 (Thermo Fisher Scientific) transfection of 293FT cells (purchased from Invitrogen; cells maintained as previously described in ref. 27) at ~50 to 60% confluence with 900 ng of the proviral vector and 100 ng of VSV-g. Media containing VLPs (“viral media”) were collected 2 d posttransfection. Viral media were then frozen at −80 °C for a minimum of 1 h to lyse cells, briefly thawed in a 37 °C water bath, and then precleared by centrifugation at 3,000 × g for 5 min. The supernatant was collected and added to fresh HEK293FT cells (6-well format) at low multiplicity of infection to prevent infection saturation. Infected cells were collected, washed with phosphate-buffered saline (PBS; 137 mM NaCl, 2.7 mM KCl, 10 mM Na_2_HPO_4_, and 1.8 mM KH_2_PO_4_), and treated with 10 mM TrypLE Express Enzyme (Gibco/Thermo Fisher Scientific). Cells were then resuspended in PBS, and 10% paraformaldehyde was added to a final concentration of 5%. After 10- to 20-min incubation at room temperature, the cells were collected by centrifugation at 500 × g for 5 min and resuspended in 500 μL PBS. Cells were assayed for fluorescence using an Attune Flow Cytometer with proprietary Attune collection and analysis software (Thermo Fisher Scientific).

Cell lysis and preparation of released VLPs were carried out as previously described (27). VLPs were concentrated over 500 μL of 20% sucrose in PBS at 50,000 × g for 45 min (Beckman Coulter Optima TLX). Infections were assessed by western blot using the following antibodies: RbαHIV-p24 (provided by the HIV Reagent Program), AlexaFluor680-conjugated GtαRb-IgG (Life Technologies, A21076), and AlexaFluor790-conjugated MsαGAPDH (Santa Cruz Biotech, sc-365062). Blots were imaged using an Odyssey Imaging System (Lambda Instruments Corporation), and band densities were measured using Image Studio Lite software (Li-COR). Raw band densities were imported into Microsoft Excel, and infections were normalized to percentage of infections for wild-type virus, followed by normalization to a GAPDH loading control. Normalized data were then exported to CSV format for ANOVA and graph generation in RStudio (53, 54).

### Negative Stain EM of CLPs.

First, 4 µL sample was applied to a glow-discharged grid (Electron Microscopy Sciences Formvar/Carbon 200 Mesh) for 30 s. Then, the grid was washed with 100 μM IP_6_ in 50 mM MES (pH 6.2 or 7.4) for ~5 s, stained with 2% uranyl acetate for 2 min, and blotted dry. Samples were imaged at 120 kV (FEI Tecnai 12 BioTwin TEM) or 100 kV (FEI Morgagni TEM).

### Cryo-ET and SPA Cryo-EM Sample Preparation.

CA CLPs (3 to 4 μL sample) were applied to a glow-discharged grid (Protochips 2/2-3Cu C-Flat grids; PELCO easiGlow glow discharger), then blotted for 2 to 3 s, and plunge-frozen in liquid ethane. Plunging was performed at 90 to 100% humidity via automatic backside blotting (via Leica Microsystems Cryo-GP 2 plunge freezer) or dual-side blotting (Mark IV FEI/Thermo Fisher Scientific Vitrobot). CLP samples for cryo-ET contained BSA-coated 10-nm colloidal gold at a CLP:gold bead ratio of ~8:1.

Liposome-templated CA (3.5 μL sample) was applied to a glow-discharged grid (QUANTIFOIL 300 mesh Au 1.2/1.3) and then manually wicked away from the edge of the grid with a Kimwipe (Kimberly-Clark). An additional 3.5 μL sample was applied, then automatically dual-side blotted for 3 s, and plunge-frozen in liquid ethane at 95% humidity/4 to 6 °C on a Mark IV FEI/Thermo Fisher Scientific Vitrobot.

### Cryo-ET and SPA Cryo-EM Data Collection.

Cryo-ET samples were imaged at 300 kV on a Titan Krios TEM (Thermo Fisher Scientific) equipped with a K2-XP direct detector (Gatan, Inc.) and a BioQuantum postcolumn energy filter (Gatan Inc.) with a slit width of 20 eV. Imaging was done at a nominal magnification of 105,000× with a physical pixel size of 1.379 Å/pixel. Using SerialEM (55), dose-symmetric tilt series (56) were collected with a tilt angle range between −60° and 60°, 3° increment, and dose fractionation of each tilt image into 10 frames.

SPA samples were imaged at 200 kV on a Talos Arctica TEM (Thermo Fisher Scientific) equipped with a K3 direct detector (Gatan, Inc.) and a BioQuantum energy filter (Gatan Inc.) with a slit width of 20 eV. Imaging was done at a nominal magnification of 63,000× in superresolution mode with a physical pixel size of 1.31 Å/pixel. Using SerialEM (55), a total of 50 frames were captured as movies. See *SI Appendix*, Table S1.

### Cryo-ET and SPA Cryo-EM Data Processing.

See *SI Appendix*, Table S1.

Cryo-ET and STA were performed essentially as previously described (5, 57). Full details are provided in *SI Appendix*.

SPA image processing was done in RELION 4.0 (58) [maintained by SBGrid (59)] and CryoSPARC (60), and motion correction and CTF estimation were carried out using MOTIONCOR2 (61) and GCTF (62). The initial map used for subsequent alignments was generated by aligning CLP particles against EMDB-3465 (5) low pass filtered to 40 Å. Structures were determined using the pipeline described in *SI Appendix*, Fig. S2.

### Atomic Model Building and Refinement.

An initial reference model was prepared in UCSF Chimera (63) by docking the crystal structure of full-length hexameric CA (PDB 4XFX) into the cryo-EM map of SUV-templated CA lattice prepared at pH 7.4 with IP_6_ and then refined using the Real Space Refinement tool in Phenix (64). The model was adjusted using the ISOLDE UCSF ChimeraX plugin (65, 66), followed by iterative rounds of manual adjustments in Coot (67) and Real Space Refinements in Phenix (64). The resulting model was used as an initial model for all other modeled structures, which were also prepared as described above. Model quality was evaluated using MolProbity (68). Cryo-EM maps have been deposited into the EMDB, and atomic models have been deposited into the PDB. See *SI Appendix*, Table S1. When preparing figures containing atomic models, IP_6_ and GS-6207 were docked according to best cross-correlation with the cryo-EM density and are not included in the atomic models. RMSD measurements for structure comparisons were performed using the MatchMaker tool in ChimeraX (66).

## Supplementary Material

Appendix 01 (PDF)

## Data Availability

Cryo-EM maps have been deposited into the Electron Microscopy Data Bank: EMD-16698 (CLP pentamer STA) (69), EMD-16699 (CLP hexamer STA) (70), EMD-29772 (lattice from CLPs) (71), EMD-29773 (templated lattice prepared with IP_6_ at pH 6.2) (72), EMD-29774 (templated lattice prepared with IP_6_ at pH 7.4) (73), EMD-29775 (templated lattice prepared with dNTPs at pH 7.4) (74), EMD-29776 (templated lattice prepared with IP_6_ at pH 7.4; bound to GS-6207/lenacapavir) (75), and EMD-29777 (templated lattice prepared in the absence of polyanion) (76). Atomic coordinates have been deposited into the Protein Data Bank: PDB 8G6K (lattice from CLPs) (77), 8G6L (templated lattice prepared with IP_6_ at pH 6.2) (78), 8G6M (templated lattice prepared with IP_6_ at pH 7.4) (79), 8G6N (templated lattice prepared with dNTPs at pH 7.4) (80), and 8G6O (templated lattice prepared with IP_6_ at pH 7.4; bound to GS-6207/lenacapavir) (81). All other data are included in the main article and/or in *SI Appendix*.
