# Supplementary material for: Structural insights into HIV-1 polyanion-dependent capsid lattice formation revealed by single particle cryo-EM
Source: Proc Natl Acad Sci U S A. 2023 Apr 24;120(18):e2220545120. doi: 10.1073/pnas.2220545120 (PMC10160977; doi:10.1073/pnas.2220545120)
Supplement: Supplementary file 1 — Appendix 01 (PDF) [file pnas.2220545120.sapp.pdf]

PNAS

Supporting Information for

Structural insights into HIV-1 polyanion-dependent capsid lattice formation revealed by single particle cryo-EM

Carolyn M. Highland[1,2], Aaron Tan[3,#], Clifton L. Ricaña[1], John A.G. Briggs[3,4], Robert A. Dick[1]*

[1] Department of Molecular Biology and Genetics, Cornell University, Ithaca, NY 14853

[2] Weill Institute for Cell and Molecular Biology, Cornell University, Ithaca, NY 14853

[3] Structural Studies Division, Medical Research Council Laboratory of Molecular Biology, Cambridge CB2 0QH, UK

[4] Department of Cell and Virus Structure, Max Planck Institute of Biochemistry, 82152 Martinsried, Germany

[#] Present address: Programme in Emerging Infectious Diseases, Duke-NUS Medical School, Singapore 169857

*Corresponding author: Robert A. Dick

Email: rad82@cornell.edu

This PDF file includes:

Table S1 (with legend)
Figures S1-S5 (with legends)
Supporting text - Methods
References

TABLE S1. Cryo-EM data collection, processing, and model validation statistics

CLP, capsid-like particle; Cryo-ET, cryo-electron tomography; EMDB, Electron Microscopy Data Bank; FSC, Fourier shell correlation; Hex, hexamer; Pent, pentamer; PDB, Protein Data Bank; RMSD, root-mean-square deviation; SPA, single particle analysis; Temp, SUV-templated

		Cryo-ET		SPA					
		CLP hex	CLP pent	CLP pent	Temp pent	Temp pent	Temp pent	Temp pent	Temp pent
**Sample details**	Sample pH	6.2	6.2	6.2	6.2	7.4	7.4	7.4	7.4
	IP6	+	+	+	+	+		+	
	dNTPs						+		
	GS-6207							+	
**Data collection**	Microscope	Titan Krios	Titan Krios	Talos Arctica	Talos Arctica	Talos Arctica	Talos Arctica	Talos Arctica	Talos Arctica
	Detector	Gatan K2-XP (Gatan)	Gatan K2-XP (Gatan)	K3 (Gatan)	K3 (Gatan)	K3 (Gatan)	K3 (Gatan)	K3 (Gatan)	K3 (Gatan)
	Energy filter	BioQuantum (Gatan)	BioQuantum (Gatan)	BioQuantum (Gatan)	BioQuantum (Gatan)	BioQuantum (Gatan)	BioQuantum (Gatan)	BioQuantum (Gatan)	BioQuantum (Gatan)
	Energy filter slit width (V)	20	20	20	20	20	20	20	20
	Nominal magnification	105,000x	105,000x	63,000x	63,000x	63,000x	63,000x	63,000x	63,000x
	Voltage (kV)	300	300	200	200	200	200	200	200
	Total dose (e-/Å^2)	123	123	50	50	50	50	50	50
	Super-resolution mode?	no	no	yes	yes	yes	yes	yes	yes
	Acquisition software	SerialEM	SerialEM	SerialEM	SerialEM	SerialEM	SerialEM	SerialEM	SerialEM
	Tilt angle range, step	−60°/60°, 3° (dose-symmetric)	−60°/60°, 3° (dose-symmetric)	-	-	-	-	-	-
	Defocus range (μm)	-1.5 to -4.5	-1.5 to -4.5	-0.6 to -1.6	-0.6 to -1.6	-0.6 to -1.6	-0.6 to -1.6	-0.6 to -1.6	-0.6 to -1.6
	Pixel size (Å)	1.379	1.379	1.31	1.31	1.31	1.31	1.31	1.31
	Frames per tilt or movie	10	10	50	50	50	50	50	50
	Number of tomograms or movies	66	66	1,487	3,284	1,799	2,646	3,037	696
**Processing**	Final number of particles	539,700	26,220	81,428	102,495	84,603	58,577	267,942	11,558
	Symmetry imposed	C6	C5	C5	C5	C5	C5	C5	C5
	B factor used for map sharpening (Å)	-230	-400	-98	-100	-80	-90	-103	-362
	Map resolution at 0.143 FSC (Å)	3.9	6.2	3.6	3.3	3.1	3.5	3.1	7.1
	EMDB ID	EMD-16699	EMD-16698	EMD-29772	EMD-29773	EMD-29774	EMD-29775	EMD-29776	EMD-29777
**Atomic model**	Protein residues			1,379	1,379	1,379	1,379	845	-
	MolProbity score			1.28	1.17	0.99	1.35	1.26	-
	Clash score			3.50	3.26	2.15	4.48	3.87	-
	Rotamer outliers (%)			0.09	0.09	0.43	0.09	0.00	-

**Atomic model (continued)**	Ramachandran favored (%)	97.26	97.78	98.00	97.34	97.58	-
	Ramachandran allowed (%)	2.74	2.22	2.00	2.66	2.42	-
	Ramachandran outliers (%)	0	0	0	0	0	-
	Ramachandran Z-score	1.66	1.79	0.87	1.91	1.88	-
	RMSD, bond length (Å)	0.004	0.004	0.004	0.004	0.004	-
	RMSD, bond angles	0.698	0.724	0.708	0.739	0.693	-
	PDB ID	8G6K	8G6L	8G6M	8G6N	8G6O	-

Table S1

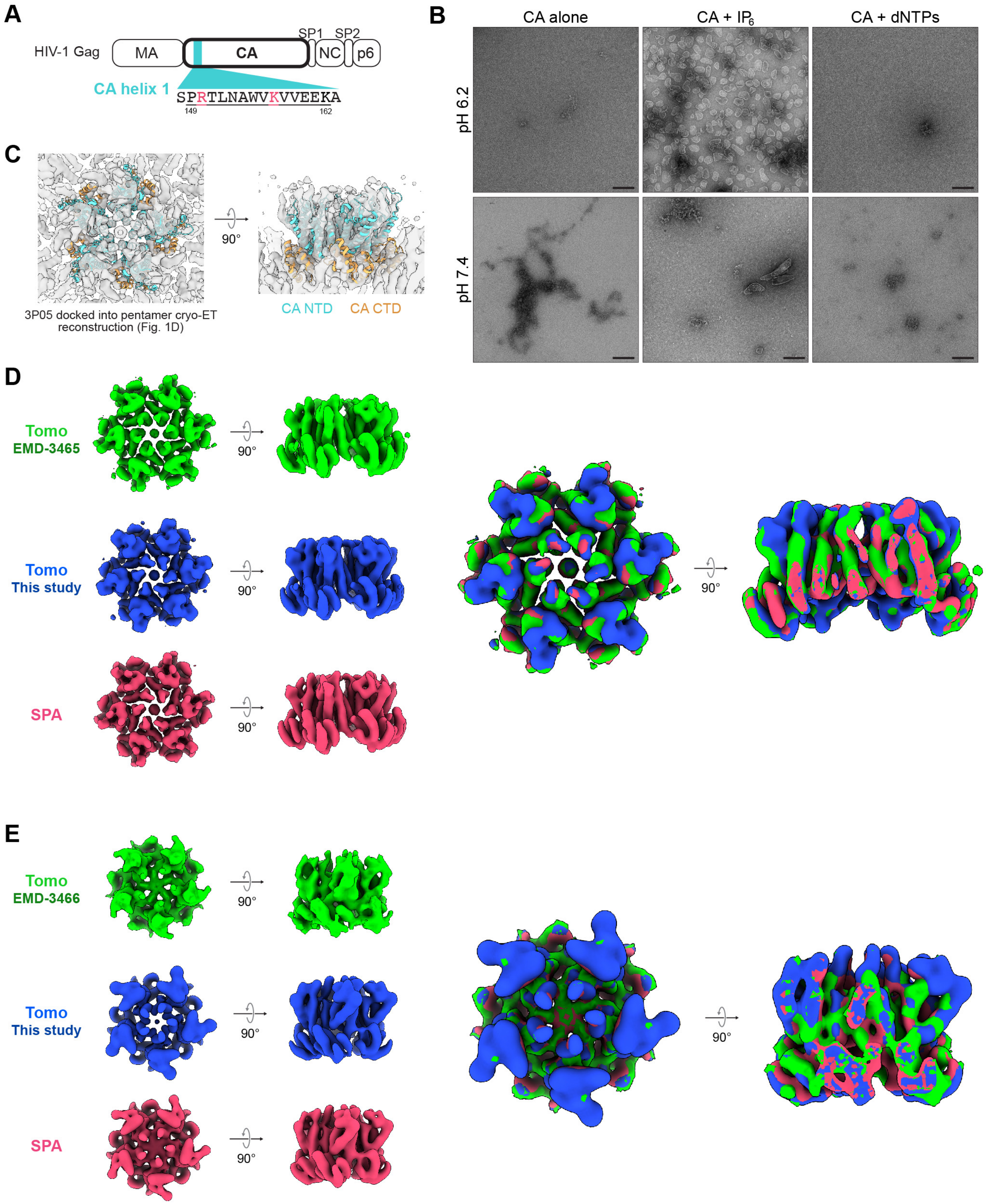

FIGURE S1. *in vitro* assembly of HIV-1 CA capsid-like particles for cryo-EM analysis. A. Domain diagram of full-length Gag, with CA α-helix 1 highlighted in blue. Gag residues R150 and K157 (corresponding to CA residues R18 and K25, respectively) are highlighted in magenta. B. Negative stain TEM micrographs of *in vitro* CLP (capsid-like particle) assembly reactions containing purified CA and polyanions at pH 6.2 or 7.4. Scale bars, 100 nm. C. Cryo-ET reconstruction of the CLP pentamer (Figure 1D) (EMD-16698) with crystal structure 3P05 (Pornillos et al., 2011) docked according to best cross-correlation with the cryo-EM density. The CA NTD is shown in cyan and the CA CTD is shown in orange. D-E. Comparison of CLP hexamer (D) and pentamer (E) maps from cryo-ET (previous structures from Mattei et al., 2016, green; structure from present study, blue) and single particle analysis cryo-EM (magenta). Hexamer cryo-ET map from this study, EMD-16699; pentamer cryo-ET map from this study, EMD-16698; SPA map from this study, EMD-29772. Maps from this study are shown low pass filtered to the resolutions of the previously published maps (6.8 Å for EMD-3465; 8.8 Å for EMD-3466). Tomo, tomography; SPA, single particle analysis.

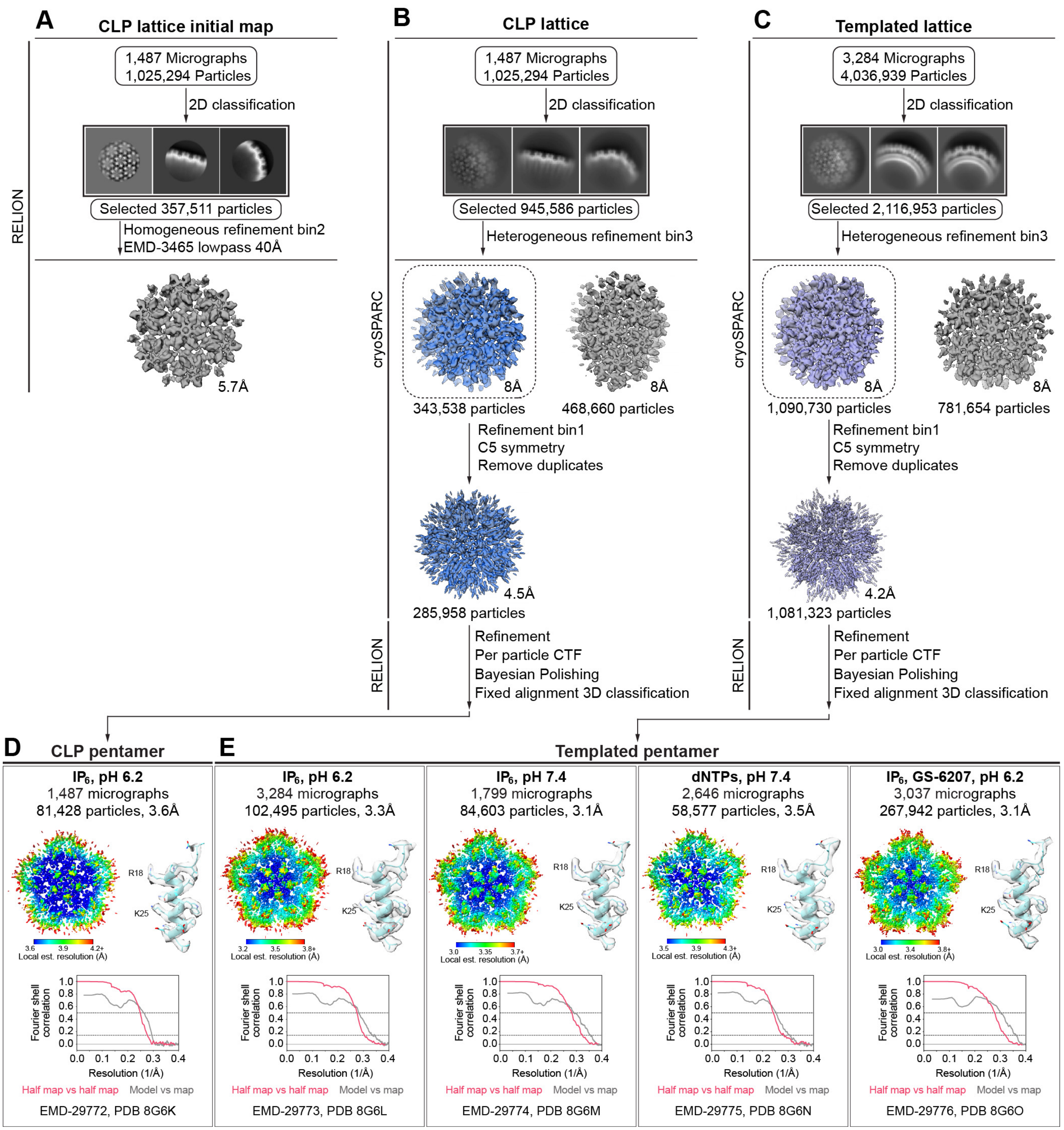

FIGURE S2. Single particle analysis data processing strategy and cryo-EM map quality. A. Preparation of initial reference for data processing detailed in B-C. B-C. Data processing strategy for 'global' pentamer structures (a central pentamer surrounded by its five nearest hexamer neighbors) from capsid-like particle (CLP) (B) and templated (C) CA lattice assembles. For C, the specified numbers of micrographs and initial extracted particles are for templated lattice prepared with IP_6 at pH 6.2. See Table 1 for data collection and processing details for all samples. D-E. Half map vs half map and model vs map Fourier shell correlation (FSC) plots and example cryo-EM densities for SPA cryo-EM maps and models from CLP (D) and liposome-templated (E) CA lattice assemblies. 0.143 and 0.5 thresholds in FSC plots are marked with dotted lines. Final particle numbers and resolutions at FSC threshold of 0.143 are listed at the top of each box. Example cryo-EM densities show residues 13-31 in pentamer CA α-helix 1.

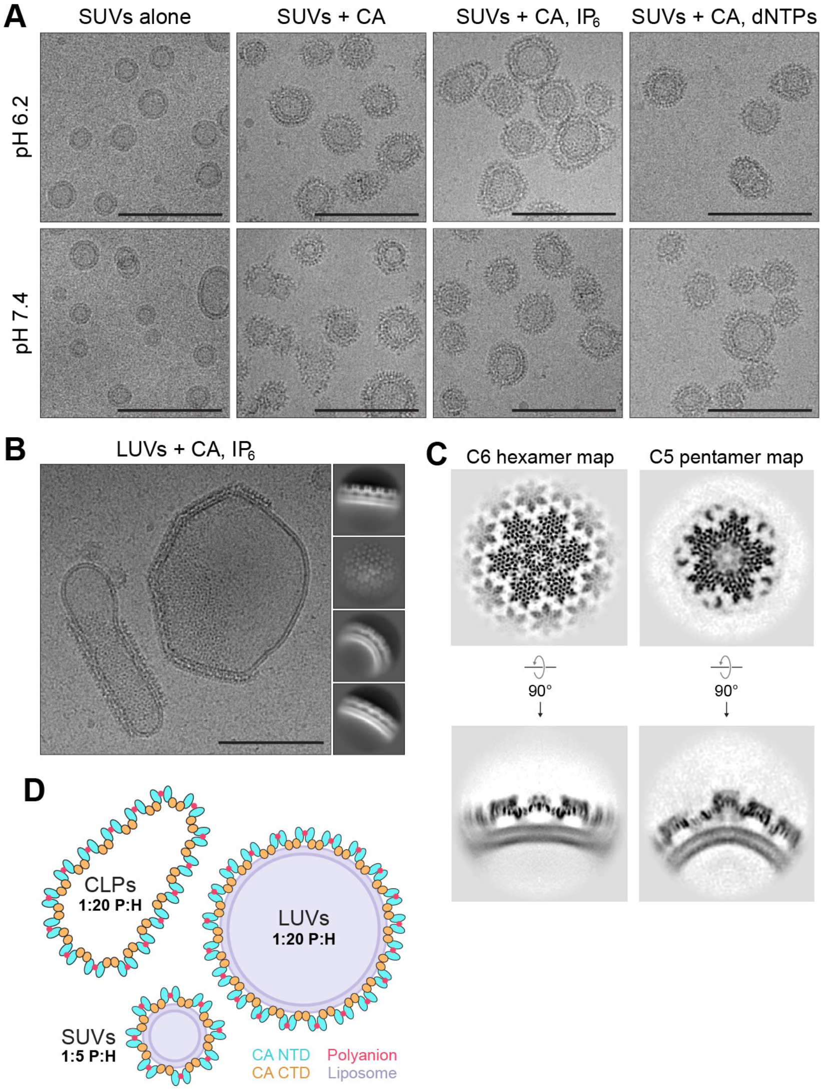

FIGURE S3. Templating HIV-1 CA lattice on SUVs enriches for pentamer formation when polyanions are present, and the pentamer imposes strict local curvature on the lattice. A. Cryo-EM micrographs of small unilamellar vesicles (SUVs) alone or with bound CA-6xHis in the absence or presence of polyanions at pH 6.2 or 7.4. Scale bars, 100 nm. B. Cryo-EM micrograph of CA-6xHis templated on large unilamellar vesicles (LUVs) at pH 6.2 in the presence of IP_6. Scale bar, 100 nm. C. Top (top) and side (bottom) orthoslice views of hexamer and pentamer maps from LUV-templated CA-6xHis lattice prepared at pH 6.2 in the presence of IP_6. Density is shown in black to differentiate it from 2D class averages. D. Comparison of typical observed pentamer-to-hexamer ratios in capsid-like particle (CLP), LUV-templated, and SUV-templated CA lattice assemblies. P, pentamer; H, hexamer.

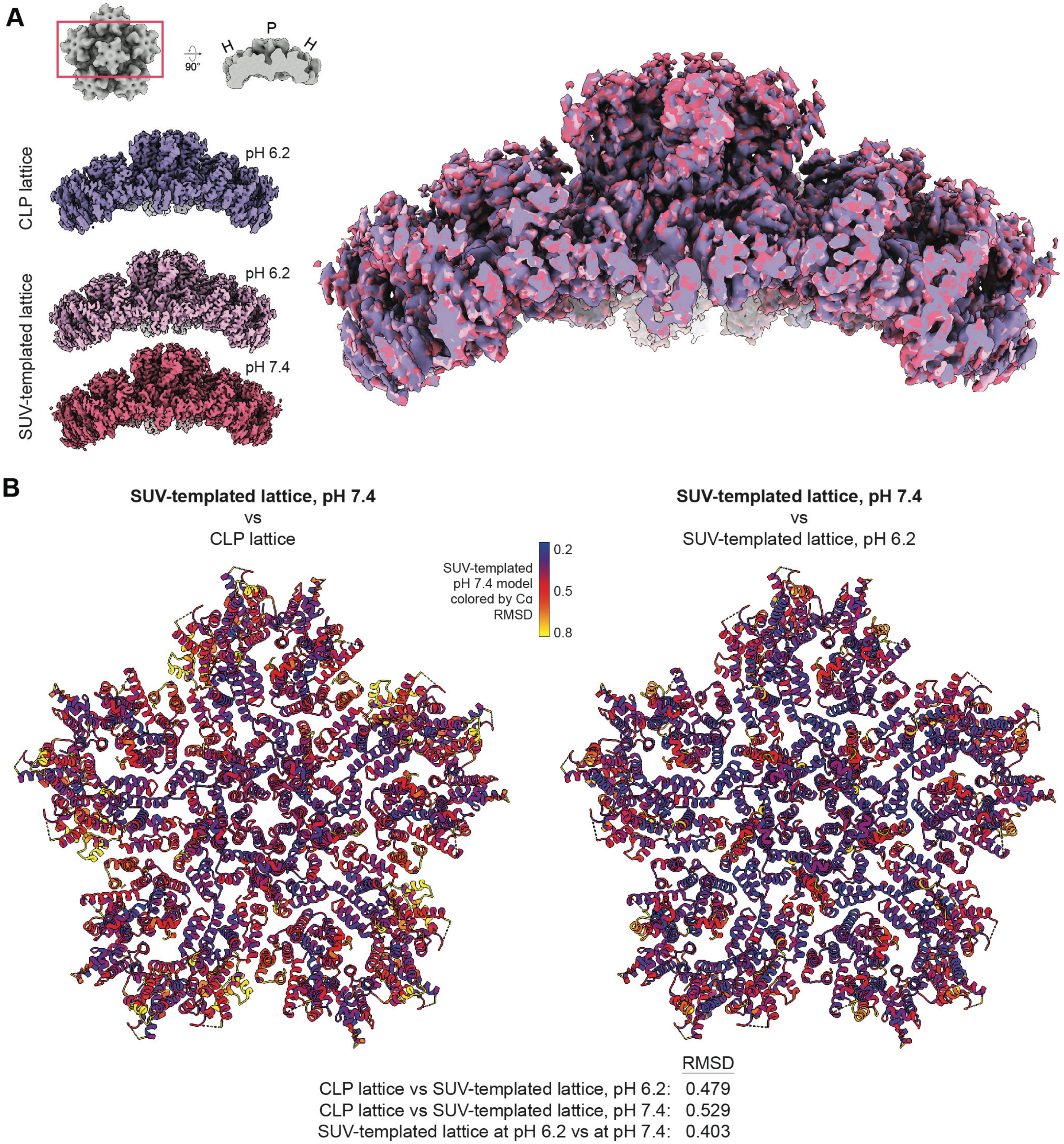

FIGURE S4. Comparison of CLP and templated CA lattice structures. A. Comparison of 'global' pentamer cryo-EM maps for capsid-like particle (CLP) lattice (purple) (EMD-29772) and small unilamellar vesicle (SUV)-templated lattice prepared at pH 6.2 (lavender) (EMD-29773) or pH 7.4 (magenta) (EMD-29774) A. Left: Top, diagram of map side views shown beneath. 'H,' hexamer; 'P,' pentamer. Beneath, individual side views of each cryo-EM map. All were assembled with IP_6. Right: Superimposed maps. B. Top, Model for SUV-templated lattice at pH 7.4 (PDB 8G6M) shown colored by Cα root-mean-square deviation (RMSD) relative to CLP lattice (left) (PDB 8G6K) and SUV-templated lattice prepared at pH 6.2 (right) (PDB 8G6L). Bottom, Cα RMSD comparisons among models for CLP lattice, SUV-templated lattice at pH 6.2, and SUV-templated lattice at pH 7.4.

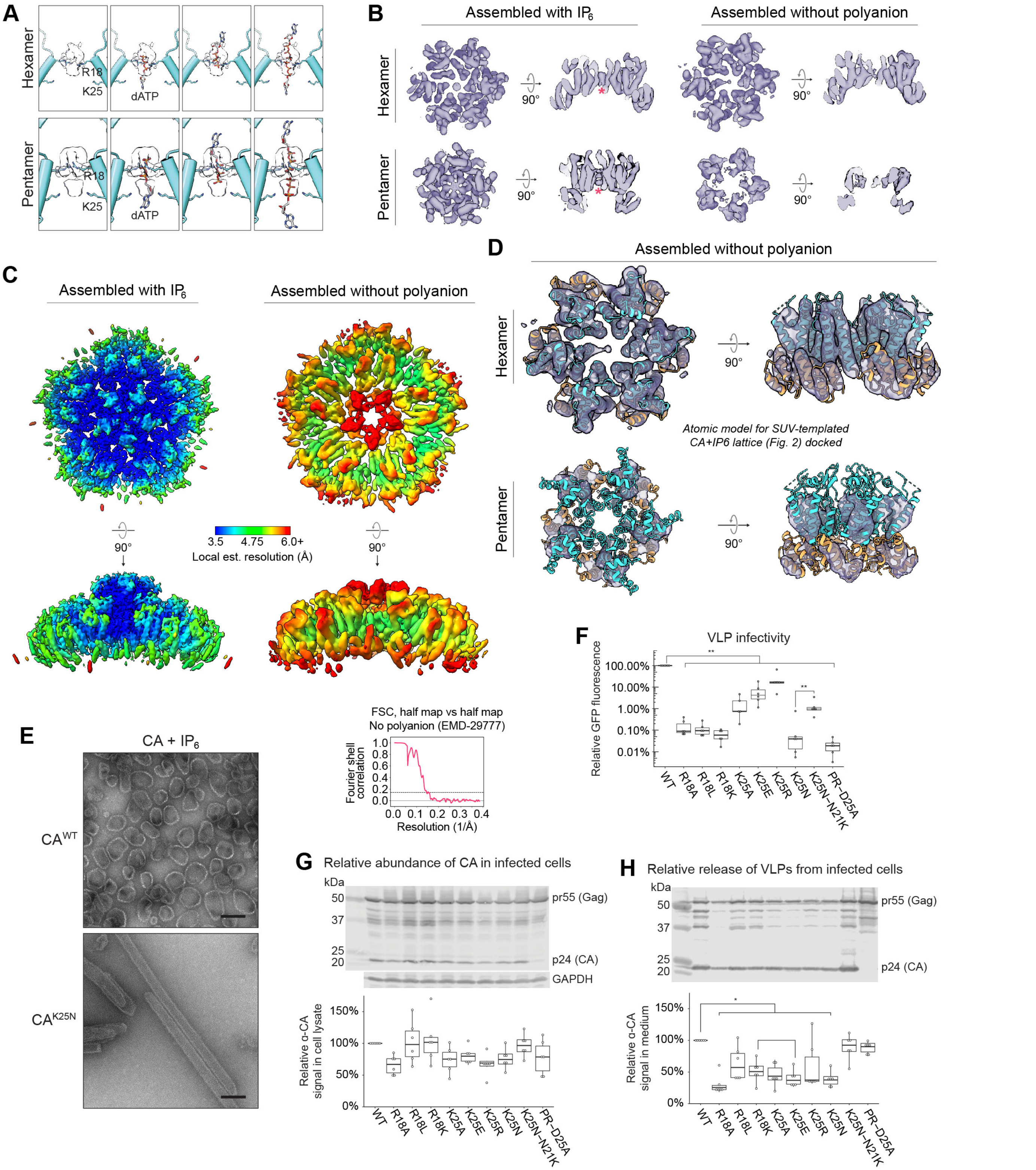

FIGURE S5. Polyanion binding is critical for proper HIV-1 CA pentamer formation and for virus-like particle infectivity and release from infected cells. A. Hypothesized dATP orientations in CA pentamer (top) and hexamer (bottom) pores from structures shown in Figure 3B,D (EMD-29775, PDB 8G6N). dATP molecules shown are manually docked into the cryo-EM density to illustrate hypothesized orientations in the pore. B. Cryo-EM maps of hexamer and pentamer alone from 'global' pentamer maps shown in Figures 3E (left) and 3F (right). Magenta asterisk marks IP_6 density. Side views show central pore cross-sections. "Assembled with IP6" map, EMD-29774; "Assembled without polyanion" map, EMD-29777. C. Cryo-EM maps from Figure 3E-F and Figure S5B (SI Appendix) colored by local resolution. Bottom right: FSC plot for the no-polyanion structure. D. Model for templated HIV-1 CA-6xHis lattice prepared at pH 7.4 in the presence of IP_6 (presented in Figure 2) (PDB 8G6M) docked into the "Assembled without polyanion" cryo-EM density shown B. E. Negative stain TEM micrographs of wild-type (WT) and K25N mutant CA assembled *in vitro* at pH 6.2 with IP_6. Scale bars, 100 nm. F. Relative infectivity of CA mutants, measured by GFP reporter fluorescence in infected cells. Data are normalized to GAPDH from the western blot shown in G. G. Top: representative western blots of HIV CA and GAPDH in infected cell lysates. Bottom: quantification of biological replicates. CA band intensity measurements are normalized to GAPDH. H. Top: representative western blots of HIV CA and GAPDH in virus-like particles released from infected cells. Bottom: quantification of biological replicates. CA band intensity measurements are normalized to GAPDH (shown in G). Statistics: N = 6, pairwise comparisons using Wilcoxon rank sum test, * $p < 0.05$, ** $p < 0.01$. VLPs, virus-like particles.

SUPPORTING TEXT – METHODS

Cryo-ET data processing – detailed methods

Pre-processing and tomogram reconstruction

Gain correction, motion correction and dose-reweighting were performed on the raw tilt series movies using alignframes from the IMOD package (1) The defocus and angle of astigmatism of each tilt image was estimated using ctfplotter from IMOD. Tilt series were manually aligned using gold fiducials in IMOD, and CTF-multiplied tomograms were reconstructed using novaCTF (2). The reconstructed tomograms were then serially binned by factors of 2, 4 and 8 in IMOD to produce binned tomograms for use in the initial stages of subtomogram alignment.

Segmentation of HIV-1 CA CLPs from tomograms

8× binned, non-CTF corrected tomograms were imported into the Ilastik software package (version 1.3.2rc1) (3) to segment the CLP cores. The vertex coordinates of the segmented volumes were used to define an isosurface for each CLP in MATLAB and an oversampled grid of points with 3.3 nm spacing was defined for subtomogram extraction along this surface using the TOM toolbox (4) with an initial estimate of the appropriate Euler angles based on the normal vector to the surface at each point.

Initial hexamer reference construction

An initial reference was constructed using a 4-tomogram subset of the CA CLP dataset containing 35 CLPs. This subset was chosen to evenly sample the range of defocus values across the data set. Subtomograms were extracted from 8× binned, 3D CTF corrected tomograms at the sampled positions with a box size of 706 Å^3. The extracted subtomograms were averaged to obtain a starting reference.

Alignment of full hexamer data set

The initial hexamer reference constructed was used as a starting reference. Subtomograms were extracted for the entire data set and subjected to iterative alignment and averaging using subTOM (link: https://github.com/DustinMorado/subTOM) based on the TOM (4), AV3 (5) and Dynamo (6) packages. After initial alignment (5° increment, 35 Å low- pass filter, C6 symmetry imposed), duplicate and misaligned points were removed, and a further iteration of alignment was performed with a 2° increment. The aligned coordinates were scaled for re-extraction from 4× binned tomograms with box dimensions of 353 Å^3. Two iterations of alignment were performed with a search range of 5 × 2° for all Euler angles, C6 symmetry and a 32 Å low pass filter. Points that had aligned onto the same positions during this iteration were removed. Subtomogram positions were scaled for re-extraction from 2× binned tomograms, with box dimensions of 353 Å^3. Data sets were split into odd- and even-numbered half-sets of equal size. The averages of each half-set were generated and all further alignments were done independently on the two half-datasets. After two further alignment iterations, positions were scaled to the unbinned pixel size, subtomograms were extracted from unbinned data with box dimensions of 264.8 Å and a series of fine alignment iterations were performed with 1° angular increment and C6 symmetry. The final map resolution was measured by gold-standard Fourier shell correlation (FSC) between the two half data sets.

Pentamer subtomogram alignment and averaging

Pentamer subtomogram positions were obtained by identifying patterns of 5 hexamers with pairwise distance constraints that satisfy pentamer geometry (7). Pentamer subtomograms were

extracted at the identified positions from 4× binned tomograms using a box size of 353 Å and averaged to generate an initial reference, which was then used as a basis for iterative alignment and averaging with a low pass filter setting of 32 Å. Misaligned pentamer subtomograms were then removed by manual inspection of the lattice maps in UCSF Chimera (8). Pentamer subtomogram positions were scaled for use with 2× binned data and were divided into independent odd and even half-sets. Subtomograms were iteratively aligned with C5 symmetry, re-extracted from unbinned tomograms with a box size of 264.8 Å and subjected to finer alignment with a 1° increment, C5 symmetry, and a low pass filter of 10.6 Å. The final map resolution was measured by gold-standard FSC between the two half data sets.

REFERENCES

1. J. R. Kremer, D. N. Mastronarde, J. R. McIntosh, Computer Visualization of Three-Dimensional Image Data Using IMOD. *Journal of Structural Biology* **116**, 71–76 (1996).

2. B. Turoňová, F. K. M. Schur, W. Wan, J. A. G. Briggs, Efficient 3D-CTF correction for cryo-electron tomography using NovaCTF improves subtomogram averaging resolution to 3.4Å. *J Struct Biol* **199**, 187–195 (2017).

3. C. Sommer, C. Straehle, U. Köthe, F. A. Hamprecht, Ilastik: Interactive learning and segmentation toolkit in *2011 IEEE International Symposium on Biomedical Imaging: From Nano to Macro*, (2011), pp. 230–233.

4. S. Nickell, *et al.*, TOM software toolbox: acquisition and analysis for electron tomography. *Journal of Structural Biology* **149**, 227–234 (2005).

5. F. Förster, O. Medalia, N. Zauberman, W. Baumeister, D. Fass, Retrovirus envelope protein complex structure in situ studied by cryo-electron tomography. *Proceedings of the National Academy of Sciences* **102**, 4729–4734 (2005).

6. D. Castaño-Díez, M. Kudryashev, M. Arheit, H. Stahlberg, Dynamo: A flexible, user-friendly development tool for subtomogram averaging of cryo-EM data in high-performance computing environments. *Journal of Structural Biology* **178**, 139–151 (2012).

7. S. Mattei, B. Glass, W. J. H. Hagen, H.-G. Kräusslich, J. A. G. Briggs, The structure and flexibility of conical HIV-1 capsids determined within intact virions. *Science* **354**, 1434–1437 (2016).

8. E. F. Pettersen, *et al.*, UCSF Chimera--a visualization system for exploratory research and analysis. *J Comput Chem* **25**, 1605–1612 (2004).
